# Evaluating the cost-effectiveness of new treatment strategies for the management of young infants with low- or moderate-mortality risk signs of possible serious bacterial infection: framework and study methodology for randomised controlled trials in six countries across Africa and Asia

**DOI:** 10.7189/jogh.16.05003

**Published:** 2026-06-26

**Authors:** Charu C Garg, Shamim A Qazi, Yasir Bin Nisar, PSBI Cost-Effectiveness Study Group, Abdullah H Baqui, Mohammod Shahidullah, Salahuddin Ahmed, Arunangshu Dutta Roy, Rasheda Khanam, Iffat Ara Jaben, Nabidul Haque Chowdhury, Kisholoy Choudhury, Sabina Ashrafee Lipi, Md Jahurul Islam, Manajjir Ali, Amha Mekasha, Lulu Muhe, Damen Hailemariam, Dorka Woldesenbet Keraga, Tabot Keskis Azeze, Abiy Seifu Estifanos, Bogale Worku, Solome Jebessa, Archana Thakur, Temsunaro Rongsen-Chandola, Nidhi Goyal, Amit Kumar, Nita Bhandari, Uma Chandra Mouli Natchu, Manisha Gupta, Aritra Guha, Shayam Kaushik, Surjeet Kumar, Amitabh Jain, Jagjit Singh Dalal, Kundan Mittal, GP Kaushal, Vineeta Wadhwa, Anju Seth, Varinder Singh, Harish Pemde, Praveen Kumar, Viswas Chhapola, Yashwant Kumar Rao, Arun Kumar Arya, Krishna Kumar Dokania, Pankaj Kumar, Ved Prakash, Amit Singh, Suryanshu Ojha, Shakal Narayan Singh, Neeraj Kumar, Shiv Kumar, Vinay Pratap Singh, Malvika Mishra, Pramod Kumar Singh, Vivek Kumar Singh, Amit Tandon, Saumya Dwivedi, Priya Chaturvedi, Madhuri Tiwari, Rashmi Kumar, Aarti Kumar, Vishwajeet Kumar, Robinson Daniel Wammanda, Laila Hassan, Ishaku Hassan, Saraja Ahmodu Opaluwa, Bawa Ega, Aminu Shadrach Adamu, Daniel Efemena Atinaya, Fyezah Jehan, Imran Nisar, Benazir Baloch, Kiran Lalani, Najeeb Rehman, Azhar Raza, Tooba Ahmed Alvi, Salman Osmani, Aneeta Hotwani, Fatimah Azhar, Karim Manji, Rodrick Kisenge, Raban Rameck, Nahya Salim, Sarah Somji, Mohamed Kheri Bakari, Fatimah Dhallah, Fred Maleko, Kristina Lugangira, Veneranda M Ndensangia, Christopher R. Sudfeld, Christopher P Duggan, Divya D Bhasin, Nikita Bindra, Karina Gupta, Chandrika Garg, Yasir Bin Nasir, Shamim A Qazi

**Affiliations:** 1 Syzygy Consulting LLC, California, USA; 2 Independent Newborn and Child Consultant, Switzerland; 3 Department of Sexual, Reproductive, Maternal, Child and Adolescent and Ageing Health, World Health Organization, Geneva, Switzerland

## Abstract

**Background:**

The World Health Organization (WHO) is coordinating two randomised controlled trials (RCTs) across three sites in Africa (Ethiopia, Nigeria, and Tanzania) and four in Asia (one in Bangladesh, two in India, and one in Pakistan) to generate evidence on the optimal place of treatment for young infants (YIs) with a single low-mortality risk sign of possible serious bacterial infection (PSBI) and switching antibiotic therapy from injectable to oral in YIs with moderate-mortality risk signs of PSBI. We present the framework and methodology used to compare the costs and evaluate the cost-effectiveness (CE) of these strategies.

**Methods:**

Cost analysis will be conducted from societal (hospital and household) perspectives. Hospital medical costs (staff, medicines, and consumables) and operational costs (inpatient bed costs, non-consumables, training, and communication) will be collected through hospital surveys. Household costs, including medical payments for treatment (registration, consultations, medications, consumables) and non-medical costs (transport, food, and wage loss), will be collected through household surveys. In both RCTs, combined hospital and household (including medical and non-medical) costs for all randomised sick YIs (SYIs) will be used, with an intention-to-treat approach, to calculate the cost per SYI in each study arm. Effectiveness measures, based on the absence of adverse outcomes, will be used to determine incremental CE ratios and incremental net benefits. Separate societal and payer/provider perspectives will be considered, and site-specific willingness-to-pay thresholds will be estimated. The household’s medical and non-medical costs, along with their share of societal costs, will estimate the household burden per treated child.

**Conclusions:**

This CE framework will evaluate thresholds for new PSBI treatment strategies in YIs, integrating health system and household perspectives. It will seek to identify safe, cost-effective approaches to reduce economic burdens, inform national budgetary impact, and potentially prompt WHO guideline revisions to improve infant care in low-resource settings globally.

In 2024, around 2.3 million neonatal deaths occurred worldwide, accounting for 47% of all under-five deaths [1], with infections responsible for up to 37% of these deaths in South Asia and sub-Saharan Africa [2]. The World Health Organization (WHO) recommends hospital referral for inpatient injectable therapy for at least seven days with supportive care, if needed, for young infants (YIs) (0–59 days old) with any sign of possible serious bacterial infection (PSBI) [3,4]. However, in resource-limited settings, a large proportion of the families of YI with PSBI refuse hospital referral due to several barriers, such as distance to the health facility, cost of hospitalisation, daily wage loss during the hospitalisation period and cultural constraints resulting in newborn deaths [5–8].

To address these barriers, several clinical trials [5–12] and research have been conducted in Africa and Asia to assess the feasibility of implementation [13] of the WHO PSBI management guidelines [14] and to establish that YI with clinically severe infection (CSI) and pneumonia (subclassification of PSBI) treated with simplified outpatient antibiotic regimens are effective. Pooled analyses of three trials from Africa and Asia reported that a simplified treatment regimen using oral amoxicillin with gentamicin had better outcomes compared to other regimens in ambulatory settings [15]. Secondary analysis of the African Neonatal Sepsis Trial further highlighted that infants with signs of CSI had higher mortality rates when treated in a hospital compared to outpatient treatment. This secondary analysis further divided these signs into low- or moderate signs based on the case fatality ratio associated with each CSI sign [16].

Cost-effectiveness analyses (CEAs) have become vital in identifying affordable and effective interventions for the management of neonatal sepsis and the reduction in neonatal mortality among vulnerable newborns. A study from Ethiopia assessed the costs of managing PSBI for YI at health posts when a referral was not feasible and found it cost-effective [17]. Similarly, the African Neonatal Sepsis Trial found that management of PSBI with simplified antibiotic regimens was cost-effective in the Democratic Republic of Congo, Kenya, and Nigeria [18]. A systematic review across 14 low-income countries estimated an average hospitalisation cost for YI with sepsis (considering both provider and payer perspectives) at USD 55 [19]. Another multi-centric implementation research in India estimated USD 18.5 as the health system cost for recommended and acceptable outpatient treatment of PSBI with simplified antibiotic regimens [20].

We present a comprehensive cost-effectiveness (CE) methodology for two randomised controlled trials (RCTs) evaluating new treatment strategies for managing PSBI in YIs. The first trial [21] will evaluate the appropriate place of treatment for YI with a single low-mortality-risk PSBI sign, and the second [22] will evaluate the duration of hospitalisation for those with moderate-mortality-risk PSBI signs by switching from injectable to oral antibiotic therapy. Additionally, this framework will provide a methodology to calculate provider and household costs across each study arm, offering a holistic perspective on the economic and clinical impacts of these interventions. The primary objective will be to determine whether the intervention is at least non-inferior to the control in preventing poor clinical outcomes in both RCTs. Additionally, it will use an incremental net benefit (INB) approach to estimate country-level willingness to pay thresholds.

## METHODS

### Study design and participants

We followed the CHEERS 2022 guidelines for economic evaluations (Checklist S1 in the **Online Supplementary Document**) and a trial-based CE framework [23]. The study involves two individual, parallel-arm, open-label RCTs being conducted at 31 hospitals at seven sites in six countries – three sites in Africa (Ethiopia, Nigeria, and Tanzania) and four sites in Asia (Bangladesh, two sites in India, and Pakistan) [21,22]. The study participants will be YI with low- or moderate-mortality-risk PSBI signs that seek treatment from selected hospitals in the study area and live within a catchment area where a follow-up up to day 15 can be accomplished.

### Interventions and controls in the two RCTs

The RCT1 is evaluating whether experimental outpatient treatment (intervention) for YI with a single low-mortality-risk PSBI sign (*i.e.* body temperature ≥38°C, severe chest indrawing, or fast breathing of ≥60 breaths/min in 1–6-day-old infants) with injectable gentamicin (once daily) for two days and oral amoxicillin (twice daily) for seven days can be as effective as the recommended care with injectable ampicillin (2–4 times daily) and injectable gentamicin (once daily) with supportive care for seven days (control) [21]. The RCT2 is evaluating hospitalised YI with moderate-mortality-risk PSBI signs (*i.e.* body temperature <35.5°C, movement only when stimulated, or stopped feeding well), alone or in combination, or multiple low-mortality risk PSBI signs. These infants are treated similarly for the first two days with injectable ampicillin (2–4 times daily, depending on the weight of the YI) and injection of gentamicin (once daily). At 48 hours after starting treatment, if they show clinical improvement in terms of the absence of all signs of critical illness or CSI, and have a negative C-reactive protein (CRP) laboratory test, they are randomised into two groups: one is discharged on oral amoxicillin (twice daily) for five days at home (intervention), and the second group continues injectable antibiotic therapy in the hospital for five more days (control) [22].

### Outcomes

Poor clinical outcomes for RCT1 [21] are death at any time from randomisation up to day 15 of initiation of treatment, any sign of critical illness (*i.e.* no movement at all, unable to feed at all, or convulsions) or any sign suggestive of another serious infection (*e.g.* meningitis, bone or joint infection on day two, four, or eight post-randomisation, any new CSI sign on day four or eight post-randomisation, or persistence of the presenting CSI sign on day eight post-randomisation).

Poor clinical outcomes for RCT2 [22] are death between randomisation (day three of initiation of therapy) and day 14 of initiation of therapy, presence of any sign of critical illness (*i.e.* no movement at all, unable to feed at all, or convulsions) or any sign suggestive of another serious infection (*e.g.* meningitis, bone or joint infection on day four and eight of initiation of therapy, or presence of any sign of CSI on day eight of initiation of therapy).

The effectiveness rates will be estimated for each treatment arm for both RCTs as the proportion or percentage of the population that did not experience poor clinical outcomes. The following formula is used:

Mean effectiveness rate (E) = (positive outcomes (O) / total population (N)) × 100

Where O is the number of individuals with positive outcomes calculated as O = N − P, N is the total number of individuals in each arm of the treatment group, and P is the number of individuals who experienced poor clinical outcomes (*e.g.* death, deterioration, or other conditions described above) among N. Mean effectiveness will vary between enrolled SYI and those who received recommended treatment, as P and N would differ. The effectiveness measure (‘proportion without poor clinical outcomes’) is chosen for its direct alignment with trial endpoints and feasibility across heterogeneous sites.

### Framework for analysis

A common standardised protocol is being implemented across all study sites to ensure consistency in participant selection, interventions, and outcome assessments. The WHO coordinating team provides central training and oversight to harmonise activities, outcomes, and analytical procedures. All participating sites have extensive research experience, further supported by regular online and in-person meetings, data monitoring, and site visits by WHO monitors. The study started on 24 June 2021, and data collection was completed on 19 August 2024.

### Hypothesis/objective

For RCT1, the objective is to determine if outpatient care with injectable gentamicin (once daily) for two days and oral amoxicillin (twice daily) for seven days can be as cost- effective compared to the currently recommended management, that is, injectable ampicillin (2–4 times daily), and injectable gentamicin (once daily) with supportive care for seven days, for low-mortality-risk PSBI signs. This could significantly alleviate the burden on hospitals, and reduce the incidence of hospital-acquired infections while lowering costs for both health systems and households.

For RCT2, we compare the feasibility and CE of switching from continued inpatient care with seven days of recommended treatment using injectable ampicillin (2 − 4 times daily), and injectable gentamicin (once daily) with supportive care (control) to outpatient care (intervention) with oral amoxicillin (twice daily) for five days after a two-day hospital stay. YIs with moderate-mortality-risk PSBI are randomised into inpatient and outpatient arm after 2 days of inpatient treatment with injectable ampicillin (2 − 4 times daily), and injectable gentamicin (once daily) and a negative CRP test. If outcomes are shown to be non-inferior, and costs are lower with the intervention, then this approach would optimise the use of hospital resources and minimise household economic burden.

Collectively, the results from these trials will provide a strong foundation for revisiting and updating WHO PSBI management guidelines, ensuring that treatment strategies align with resource-constrained settings while maintaining clinical safety [24].

### Costs framework

The cost will be assessed from both the hospital and household perspectives. From the hospital perspective, medical treatment costs will be determined at each study hospital across all sites in both the intervention and control arms (**Table 1**, **Table 2**). Additionally, the household's costs will be separated into medical and non-medical costs. The hospital's indirect operational costs and households' opportunity costs in terms of wage loss will also be estimated. The costs will be weighted by infant days for each hospital, separately for the control and intervention arms, to obtain site-specific average costs for each item. To generate combined estimates, we will pool site-specific average costs across all sites to produce a single multi-country mean cost for inpatient and outpatient management, respectively.

**Table 1:** Framework for provider costs under RCT1: outpatient versus inpatient management of young infants (0–59 days) with low-mortality-risk PSBI signs

Cost category	Intervention arm: outpatient treatment	Control arm: inpatient treatment
Place of treatment	Outpatient/ambulatory care	Hospital inpatient care
Antibiotic regimen	Injectable gentamicin once daily for 2 days plus oral amoxicillin twice daily for 7 days	Injectable ampicillin (2–4 times daily depending on age) plus injectable gentamicin once daily for 7 days
Staff costs	Outpatient assessment, counselling, injections preparation (canula), gentamicin administration, follow-up visits	Inpatient assessment, nursing care, injections preparation (canula) + administration, monitoring and supportive care
Medicine costs	Gentamicin and oral amoxicillin	Ampicillin and gentamicin
Consumables	Syringes, needles, cotton swabs, gloves, cannula, spirit	Syringes, needles, intravenous supplies, cotton swabs, gloves, cannula, spirit
Inpatient bed costs	Not applicable	Included for the duration of hospitalisation. Captures costs in the paediatric wards, such as costs of non- consumables, utilities, security, and laundry
Operational and indirect costs	Training, supervision, communication, administration and programme support	Training, supervision, communication, administration and programme support
Weighting approach for pooled analysis	Infant-days (number of infants treated and days each medicine or consumable is used)	Infant-days (number of infants treated and days each medicine or consumable is used)

PSBI – possible serious bacterial infection, RCT – randomised controlled trial

**Table 2:** Framework for provider costs under RCT2: shorter versus standard hospital stay for young infants with moderate-mortality-risk PSBI signs

Cost category	Intervention arm: shorter hospital stays plus outpatient treatment	Control arm: standard inpatient treatment
Place of treatment	Initial hospitalisation for 2 days, followed by discharge home	Continued hospitalisation for 7 days
Antibiotic regimen	Injectable ampicillin plus gentamicin for 2 days, followed by oral amoxicillin twice daily for 5 days at home	Injectable ampicillin plus gentamicin for 7 days in the hospital
Eligibility for randomisation	Clinical improvement after 48 hours, negative CRP test and feasible follow-up	Not applicable
Staff costs	Initial inpatient assessment, injections preparation (canula) + administration, inpatient clinical monitoring, nursing and supportive care (for 2 days), discharge counselling, outpatient follow-up	Initial inpatient assessment, injections preparation (canula) + administration, inpatient clinical monitoring, nursing and supportive care (for 7 days), discharg
Medicine costs	Injectable antibiotics (gentamicin + ampicillin) for 2 days plus oral amoxicillin (twice daily) for 5 days	Injectable antibiotics (gentamicin+ ampicillin) for the full treatment duration
Consumables	Syringes, needles, CRP kits, cotton swabs	Syringes, needles, intravenous supplies, CRP kits, cotton swabs,
Inpatient bed costs	Indirect costs in paediatric wards, such as costs of non-consumables, utilities, security, laundry, *etc.* Included for the first 2 inpatient days	Indirect costs in the paediatric wards, such as costs of non-consumables, utilities, security, laundry, *etc.* Included for a full 7-day hospital stay
Operational and indirect costs	Training, supervision, communication, administration and programme support	Training, supervision, communication, administration and programme support
Weighting approach for pooled analysis	Infant-days (number of infants × days)	Infant-days (number of infants × days)

CRP – C-reactive protein, PSBI – possible serious bacterial infection, RCT – randomised controlled trial

The households incur out-of-pocket expenditures, which can be separated into direct medical expenses (registration/consultation, medicines, and consumables) plus other non-medical expenses (transport, food, and wage loss). For RCT2, laboratory costs for CRP tests are also included. Wage loss occurs due to loss of income due to absenteeism from work. Site-level costs per SYI will be based on the sample size under each item for both arms for each trial, but combined costs will depend on the number of enrolled infants at each site.

### Data collection

#### Hospital data

Provider data will be gathered using standardised hospital/provider questionnaires for both control and intervention arms in RCT1 and RCT2. Data will be collected from 31 hospitals (30 in RCT1 and 29 in RCT2) from all seven sites (four hospitals in Bangladesh, five in Ethiopia, six in India Himachal Pradesh and the National Capital region, six in India Uttar Pradesh, three in Nigeria, five in Pakistan, and two in Tanzania) where YI are enrolled for treatment under RCT1 and/or RCT2.

Provider data for CEA will include the following variables:

1. Coverage of sick YIs (SYI) assessed, enrolled and treated under both RCT1 and RCT2 for control and intervention.
2. Data on two outcome variables for all SYI covered under different treatment arms. Well-trained independent outcome assessor visits all enrolled cases on days two (only for RCT1), four, eight, and 15 after the initiation of treatment to assess the study outcomes in both intervention and control arms.
3. Average annual salaries of nurses, paediatricians, medical officers and any other staff supporting treatment. To calculate staff costs per minute, data will be collected on the average number of days in a month and the average number of hours in a day that staff work.
4. Staff time will be considered only for activities that are linked to hospital staff and not those linked to research staff, such as outcome assessment or treatment documentation (**Table 1**, **Table 2**). The activities for the staff time will be based on a pre-defined implementation strategy [21,22].
5. Data will be collected for SYI that receive at least 80% of the treatment (recommended treatment) and those who receive <80% of seven-day (<5.5 days) treatment (partial treatment). The latter occurs due to a treatment failure, a change in treatment, readmission, left voluntarily, or death. For the recommended treatment, we will collect costs for the seven-day treatment period following the initial assessment, across both study arms and both trials. For partial treatment, medical costs will be calculated for an average treatment period of three days in RCT1 after the initial assessment, and for two days in RCT2 in both inpatient and outpatient arms after randomisation. Costs for enrolled SYI will be weighted by the proportion of participants receiving 80% recommended treatment and those receiving partial treatment, with weights set to SYI for each group. This prevents any child randomised and enrolled from being missed. Costs due to complications, changes in treatment, and/or readmission are outside the seven-day recommended treatment window in both study arms of both trials and will not be collected.
6. Data on staff time per visit for an activity and the number of visits undertaken for each activity during the recommended treatment period will be collected by interviewing at least 3–5 staff of each type at each hospital.
7. The staff time for all visits during the treatment period will be weighted by the number of days an infant receives the activity and the number of YI receiving the activity (defined as weighted by infant days). For the first activity assessment on presentation at the hospital, the number of SYI will be all enrolled for a given treatment, whether partial or recommended. For all other activities, SYI who receive 80% recommended treatment will be considered to have received seven days of treatment, and those receiving partial treatment for an average of three days to calculate the costs for those enrolled.
8. The average exchange rate used to convert all the cost estimates in local currency into USD will be for 2024, the period for which the salaries of providers, prices and household costs will be reported. It will ensure consistency, and inflation rate adjustments will not be needed for cost calculations. The recommended treatment period will be seven days for inpatient treatment in the control arm and seven days for outpatient treatment in the intervention arm for RCT1. For RCT2, the average length of inpatient treatment is 48–72 hours, prior to a negative CRP laboratory test. In RCT2, the control group after randomisation will continue receiving inpatient care for five days, while the intervention group will be discharged from the hospital on oral amoxicillin for five days to be taken at home. Both arms will share the initial hospitalisation costs during the first two days in RCT2, including CRP test costs (including staff time, kits, and laboratory costs).
9. The data on quantities used of injection gentamicin, injection ampicillin and oral amoxicillin will be as per the recommended treatment and partial treatment per SYI for each treatment arm under each RCT.
10. The number of SYI receiving specific medicines and consumables for each arm under both RCTs will be calculated based on the numbers receiving recommended treatment and those receiving partial treatment.
11. Information on types and quantities of consumables required with each medicine will be collected separately for each hospital and each arm under both RCTs. The cost of medicines and consumables will be charged per size in which they are dispensed. Medicine dosage varies with the weight of the YI [21,22]. Price will be taken for full strength, and the rest will be marked as wastage. Price data will be taken from the hospital's pharmaceutical department (if available); otherwise, the market price or the WHO price will be used.
12. Data on annual financial expenditures and admissions in the newborn intensive care units or paediatric wards where YI with PSBI are treated as inpatients at the hospital will be collected and allocated based on patient days to calculate the inpatient bed costs/ SYI treated as an inpatient. These expenditures include administration, utilities, laundry, security, data management, internet, non-consumables and other costs in the hospital inpatient ward.
13. Hospital ambulance to transport enrolled SYI under a specific treatment arm is a cost to the hospital. It is difficult to get this information separately for each enrolled SYI under different treatment arms from the hospital data. Indirect estimation based on the average trip cost and the number of SYI using free public ambulances, estimated from household data for each treatment arm in RCT1 and RCT2, will be used.
14. Hospital data will also be collected for operational costs, which help to strengthen the assessment, identification, and treatment of SYI with PSBI. These include training, supplies, and communication. The total costs under these three will be estimated per SYI assessed. Staff training costs include the costs of development of training material and training cost per staff per day based on the per diems, honorarium for trainers, venue and refreshment costs. Total training costs will be calculated for all staff who were used to assess and treat SYI and will be estimated per SYI assessed. Training costs for research staff for outcome assessment or data collection will not be collected or included. Price and quantities of supplies used during the assessment, such as timers, thermometers and weighing scales, will be prorated for PSBI assessed. Communication costs, include job aids or educational material, and the cost of videos developed and played at the hospitals for educating the mothers about the danger signs. Data will be collected on item quantities and prices from hospitals to assess total costs and costs per SYI assessed.

#### Household cost data

1. Household expense data will be collected during the last nine months of the study at each site to capture the out-of-pocket expenditures that households incur during the treatment of enrolled SYI under both treatment arms for RCT1 and RCT2. Data will be collected from at least 10% of SYIs enrolled under each arm at each site. These sampled households will be assumed to be representative of all treated YIs both temporally and geographically. Further, the household data for the seven-day treatment period will be collected either on day eight or 15 of the outcome visit or at most within two weeks from the last outcome assessment, to avoid recall bias. Where available, caregiver-reported expenditures will be cross-checked with receipts or facility payment records to enhance accuracy.
2. Standardised household questionnaires will be used across all sites to collect data on medical and non-medical costs using the REDCap tool [25,26]. Training will be provided to collect data using these forms, and pilots will help improve the tool and data quality. Data inconsistencies will be identified, discussed with the sites and corrected at the analysis stage.
3. Data will be collected on the type of treatment arm and duration of treatment. Direct treatment costs for each component will be collected in local currency from sampled households in each treatment arm for RCT1 and RCT2 at each site. Total medical expenses by households will be collected for registration and consultation fees, expenditures on medicines such as amoxicillin, ampicillin, gentamicin and other supportive care such as paracetamol, consumable supplies such as syringes, needles, canulas, bandages, spirits, gloves, laboratory expenses such as x-ray, blood work, urine tests (investigation costs), or any other payments to hospitals. Non-medical expenses, such as transport and food, will be recorded for seven days. Data will include the mode of transportation and expenses incurred by any household member on round-trip trips during the treatment period. Similar information will be collected on food, with the amount spent outside the home during the days the infant had to stay at or visit the hospital.
4. Indirect expenses are opportunity costs, or the costs incurred by the household in terms of wage loss to take care of the sick child. Data will be collected from parents and caretakers who lost their wages due to time spent caring for the child. Household work that does not entail income will not be included in the opportunity cost. Data will be collected on the type of occupation/source of income, average earnings on a daily, monthly, or yearly basis (in the provided ranges), and the number of days not able to work to take care of the SYI. Maximum seven days of wage loss will be considered.

### Statistical analysis procedures

The statistical analysis for the hospital cost data will be done in Microsoft Excel (Microsoft, Redmond, WA, USA). The data from all facilities where infants are randomised will be entered into a structured Excel document. The average costs per SYI for staff, medicines, consumables, and inpatient bed costs, will be calculated as outlined below. Mean, median, and 95% confidence intervals will be calculated using t-values per SYI receiving recommended or partial treatment for each of the cost variables at the hospital, as well as at the household level. The medical treatment costs per SYI will be the sum of the weighted average of staff costs, medicines, consumables, inpatient bed costs, and operational costs, where weights are infant-days for those receiving recommended and partial treatment. Non-medical costs on transport, food and wage loss per SYI will be added to total medical costs per SYI to estimate the total societal costs for the control and intervention arms for RCT1 and RCT2 separately. Household payments for both medical and non-medical treatment would be added to calculate the household burden in total societal costs.

The treatment outcomes for all enrolled in the control and intervention arms will be used to estimate incremental CE ratios (ICERs) for both RCTs.

### Estimation of staff costs

**Figure 1:**
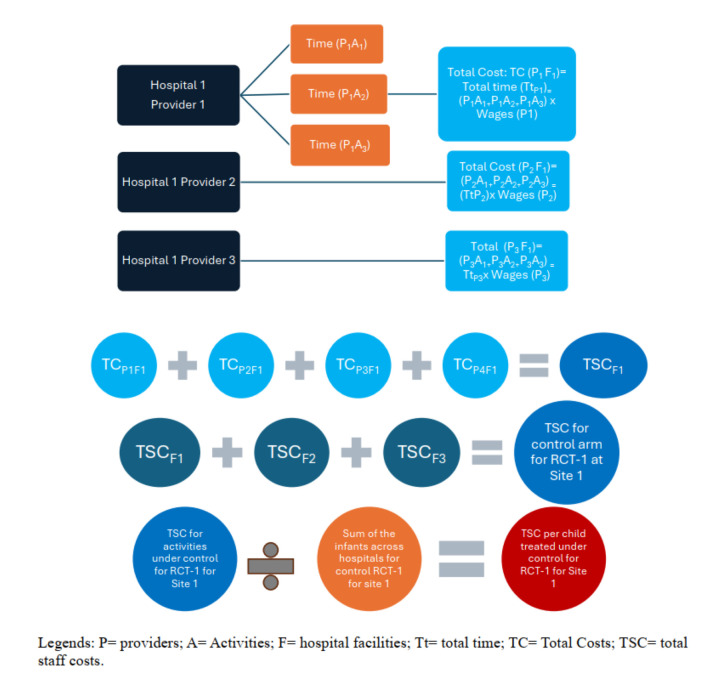
Pictorial representation of the steps for calculating staff costs per sick young infant for a given arm for a given trial. A – activities, F – hospital facilities, P – providers, TC – total costs, TSC – total staff costs, Tt – total time.

The following steps will be followed to calculate the direct staff costs per SYI at a given site (**Figure 1**):

1 Average staff time per SYI will be calculated first at each hospital for a given staff type for each activity by multiplying the time (t = minutes per visit) and the number of visits/ SYI for the activity during the treatment period (V). The number of visits for an activity per SYI can range from one visit for assessment on admission to 21 visits for daily assessments, including vitals (generally three visits per day for 7 days of treatment). However, a given staff type, for example, one nurse may take a longer time or do more or fewer visits for an activity than another nurse at the same or another hospital. Hence, the total time (T) per SYI for each activity (A) per provider type (P) at a site under different arms and trials will be TPi = (average (ts × Vs)), where ‘i’ is for providers of the same type to get the time and visits information, and ‘s’ is for each staff member of a given provider type that is interviewed. Providers ‘i’ are nurses, medical officers/ general practitioners, paediatricians, or any other provider specified. We will take the average across providers of the same type to obtain the average time for an activity during the entire treatment episode for each arm in both RCTs.
2 Staff time per provider ‘i’, per activity ‘j’ for a given hospital/facility (F) ‘k’ across all SYI getting recommended treatment will then be calculated as staff time per provider i per activity j for a given hospital k = T × N × %, where N is the number of SYI receiving activity for full days of recommended treatment (N), and % is the percentage of infants treated by a given provider for the given activity. Activity ‘j’ is described for RCT1 (**Table 1**) and RCT2 (**Table 2**) under control and interventions for staff time; ‘k’ is the hospital for which the data will be collected using the provider/hospital questionnaire.
3 The average time will be aggregated for all activities for a given provider and facility by using the number of SYI receiving recommended treatment as weights for each activity.
4 The total time for each staff will then be multiplied by the respective staff salaries/ minute (calculated based on annual salaries, the number of days worked in a year, and the number of hours worked in a day) at each hospital to obtain the staff costs/provider for treating all infants completing recommended treatment at a hospital.
5 These staff costs will be added across the providers and then across the hospitals to obtain the total staff costs at a given site.
6 These staff costs will be divided by the total number of SYI with recommended treatment under a given arm in each trial to get the staff costs/SYI for control and intervention separately for both RCTs (**Figure 1**).
7 Likewise, staff costs per SYI will be calculated for each treatment arm under both RCTs for partial treatment.

### Estimation of medicines and consumables costs

The following steps will be followed to calculate the medicines and consumable costs per SYI at a given site:

1. For each medicine used under a given arm for a given trial, the total cost of a medicine for each SYI will be calculated by multiplying the price per vial or tablet for a medicine (P) by the quantities of medicines required per SYI (Q) for recommended treatment and the number of infants receiving the medicine (n). Average medicine quantities will differ by age and weight of the SYI [21,22], and there will be associated wastage when standard-sized injectables or tablets are used. For the hospitalised SYI, injection ampicillin is given twice daily in the first week of life, thrice daily in 2–4 weeks of life, and four times daily in four or more weeks of life. 500 mg vials will be used to draw the required doses based on the SYI’s weight and age. Assuming an average dose of 200 mg three times a day, at least two vials per day will be needed for a SYI, totalling 14 vials over seven days. With a daily requirement of 600 mg, the average wastage is 40%; at higher weights, it decreases to 25%. For gentamicin, an ampoule of 40 mg/ml or 20 mg/2 ml will be used, from which 8 mg, 16 mg, or 24 mg will be administered once daily, depending on the YI’s weight. Assuming an average requirement of 16 mg, seven ampoules of 20 mg will be needed for the control arm and two ampoules for the intervention arm in both RCT1 and RCT2. This would lead to an average wastage of 20%. For amoxicillin, 14 dispersible tablets of 250 mg will be required for seven days of treatment in RCT1 and 10 dispersible tablets for five days as an outpatient in RCT2. SYI <4 kg will require half a tablet, and those weighing >4 kg will require a full tablet. We assume, on average, 14 tablets will be dispensed for each SYI. Assuming half the SYIs are <4 kg and that, on average, only half a tablet is used from those dispensed, there would be 25% wastage.
2. For each medicine at each hospital, the cost of each consumable will be calculated by multiplying the price per unit of the consumable (Pc) by the consumable quantities required per SYI (Qc) for recommended treatment with that medicine, and the number of infants receiving the consumable (n).
3. The total cost of medicines will be estimated by adding the costs of all medicines at a given hospital that are needed for each arm for each RCT.
4. Similarly, the total cost of the consumables will be estimated by adding the costs of all consumables corresponding to the medicines needed for each of the arms under the two trials at a given hospital.
5. A few consumables are not linked to specific medicines but are related to the treatment of SYI, such as a cannula fixer, a cannula for intravenous injections, gloves, and CRP kits for protein laboratory test. These costs will also be calculated by multiplying the price per unit of the consumable (Pc) with consumable quantities required per SYI (Qc) for recommended treatment with that medicine, and the number of infants receiving the consumable (n) at the hospital.
6. The total cost of consumables will be estimated by adding the costs of all consumables at a given hospital needed for each arm under the two trials.
7. The total costs of medicines and consumables (estimated separately) at each site will be calculated by adding the costs at each hospital for each arm under the two trials.
8. Average weighted medicine and consumables costs at a site will be calculated by summing the costs of all medicines and all consumables (separately) across all hospitals for each treatment arm. These total costs of medicines and consumables (estimated separately) will be divided by the number of SYI receiving recommended treatment at each hospital within a site to estimate medicine and consumable costs per SYI.
9. For partial treatment, we will follow the same procedure (in steps 1–8) as for recommended treatment. Medicines and consumables will be calculated for three days for RCT1 and for two days after randomisation for RCT2. All medicines and consumables during the first two days of inpatient treatment will be common to both the recommended and partial treatment for RCT2.

### Estimation of hospital operational costs

1. Data collected for hospital indirect cost components – training, communications and supplies – are annual and relate to all infants assessed for PSBI during that year. For each site, expenses for training, communications and supplies will be added across facilities. The number of infants assessed during a year at a hospital will be estimated based on the total number of infants assessed over the entire study period and proportionally for 12 months. The sum of expenditures for each component will be weighted by the number of SYI assessed at each hospital for PSBI during the year to obtain the weighted expense per SYI at each site.
2. Inpatient bed costs are shared costs across all infants and children admitted to newborn intensive care units and paediatric wards for shared items such as non-consumables, utilities, laundry, security, administration and other departmental expenses. The costs per admitted child will be estimated by dividing the total annual administrative expenditures (exclusive of staff costs, medicines, and consumables costs for enrolled SYI) of the paediatric and newborn intensive care units by the total number of children admitted in that year. These will be adjusted proportionately based on the average length of stay for enrolled SYI with PSBI relative to other SYI admitted during the year. We assume there are no inpatient bed costs for the intervention arm (outpatient) under RCT1 and for the 5-day ambulatory treatment in RCT2.
3. Hospital transport/ambulance costs may be incurred for some patients brought to the hospital by public ambulance. Since it is difficult to obtain the data from the hospital database for each SYI enrolled, an indirect estimation method will be used. Household survey data will be used to determine the proportion of SYI who report using free public transport under different RCT treatment arms. Each site will calculate the average cost per trip based on the average distance to the hospital and the cost per km or mile. The cost per km depends on drivers' salaries, fuel costs for the average distance to hospitals in the study area, and vehicle maintenance costs at the site. Multiplying the average costs by the estimated number of SYI using public transport under each arm will provide an estimate of transport costs incurred at the hospital level.

### Estimation of the household costs

1. Direct treatment costs reported by the households for each of the individual components will be compared, and data quality checks will be performed. Expenses incurred by households for each component will be estimated per SYI, based on the number of households in the sample after removing outliers. Outliers will be flagged using the median-absolute deviation and values exceeding plausible ranges (*e.g.* food costs exceeding World Bank *food per capita* benchmarks and wage losses based on World Bank *per capita* benchmarks).
2. Household expenses included for CEA will be for registration/ consultation, recommended medications (gentamicin, ampicillin, amoxicillin), recommended consumables (syringes + needles, cotton swabs and CRP (for RCT2)), transport, food and wage loss. All other non-recommended treatments for which households incur expenditure as part of the PSBI treatment will be reported as an additional burden.
3. For each treatment arm, total transport expenses will be added for all days (maximum seven) that transport is used by any caretaker for round-trip travel, and this is then divided by the number of sampled households after conducting the quality checks and removing outliers.
4. For food charges, a similar method of estimation will be used as for transport expenses. Total expenses reported by any caretaker during the treatment period will be summed and then divided by the number of households in the sample, after removing outliers.
5. Wage loss will be calculated per family or per SYI for each of the treatment arms.
  - Average wage/day will be calculated even when annual or monthly wages are given. The average wage for each caregiver will be the average of the range.
  - Average wage/day for each caregiver will be multiplied by the number of days lost for a SYI for any treatment. Maximum days lost by any caretaker will be capped at seven days of recommended treatment
  - Sum of the total wage loss for a family for all caretakers combined will be (A) under any treatment arm.
  - Sum of the number of families that report positive or zero wage loss for any of the caretakers will be (B). SYI where family members did not provide consent will be excluded from the denominator for calculations.
  - Total wage loss per SYI or per family under each treatment arm = A / B

All recommended or non-recommended medical and non-medical costs will be calculated per SYI as an additional burden on households.

### CEA

CE will be evaluated by estimating the ICER, defined as the difference in mean costs between the intervention and control arms divided by the difference in effectiveness between the two arms: ICER = (C_1_ − C_2_) / (E_1_ − E_2_), where C_1_ are the average costs for the intervention group; C_2_ are the average costs for the control group; E_1_ is the mean effectiveness measure for the intervention group, and E_2_ is the mean effectiveness measure for the control group. We will use societal costs – hospitals and households’ – medical costs (staff time, medicines, consumables); hospital inpatient bed cost, operational costs and households’ non-medical costs (transport, food and wage loss) for ICER calculations for all randomised SYI in both arms. We will also report ICER from only the provider/payer perspective using only medical costs for all enrolled SYI and those receiving at least 80% of the recommended seven-day treatment as a sensitivity analysis [27].

For RCT2, while the baseline costs will be presented for both pre- and post-randomisation for both study arms, the costs for ICER calculations will be compared only post-randomisation.

For each RCT, cost measures will be evaluated in both the control and intervention arms for all enrolled infants who receive either the full seven-day treatment or partial treatment. Effectiveness will be assessed in the same population based on clinical outcomes recorded on days two (for RCT1), four, eight, and 15 following treatment initiation. The ICER will be calculated for each RCT to assess whether the intervention is more cost-effective than the control.

**Figure 2:**
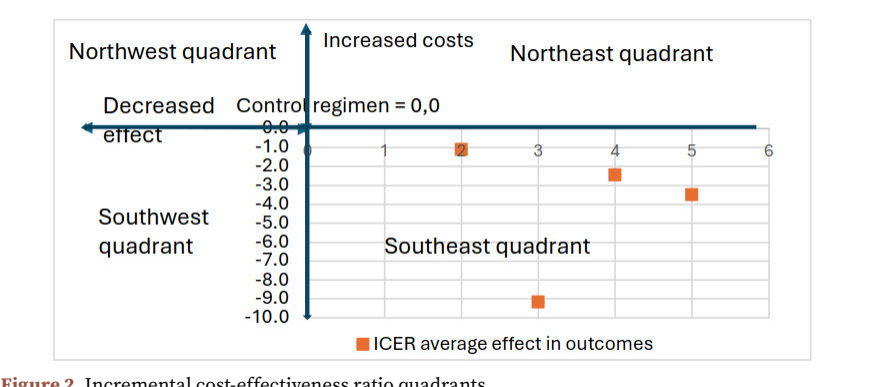
Incremental cost-effectiveness ratio quadrants.

There can be 4 case scenarios for ICER (**Figure 2**):

1. If C_1_ < C_2_ and E_1_ > E_2_, the ICER is negative and falls in the south-east quadrant of the CE plane. In this case, the intervention is less costly and more effective, indicating that the intervention dominates the control.
2. If C_1_ > C_2_ and E_1_ < E_2_, the ICER is negative and falls in the north-west quadrant of the CE plane. In this situation, the intervention is more costly and less effective, indicating that the control dominates the intervention.
3. If C_1_ > C_2_ and E_1_ > E_2_, the ICER is positive and falls in the north-east quadrant of the CE plane. Here, the intervention is more costly but also more effective. The decision regarding CE depends on the CE threshold, which may vary across countries or health systems.
4. If C_1_ < C_2_ and E_1_ < E_2_, the ICER is positive and falls in the south-west quadrant of the CE plane. In this case, the intervention is less costly but also less effective. Decisions about adoption may depend on policy considerations, such as whether a lower-cost intervention could be implemented at a larger scale to improve overall coverage and population-level effectiveness.

ICER values with negative signs are generally easier to interpret. Values in the southeast quadrant indicate that the new treatment is more effective and less costly than the existing treatment. Conversely, ICER values in the northwest quadrant indicate that the existing treatment dominates the new intervention. Positive ICER values in the southwest quadrant may suggest a cost-minimisation trade-off, but these results should be interpreted with caution, as the intervention is associated with reduced effectiveness.

In addition to ICER, we will estimate INB for each site and combined to support the interpretation of CE results [28–30]. INB are useful for quantifying uncertainty in CE, especially when differences in effectiveness are small (making ICER unstable) or when ICER results are inconclusive. For each site, incremental cost Δc = (C_1_ − C_2_) and incremental effect Δe = (E_1_ − E_2_) between intervention and control will be estimated, and INB will be calculated as λ (Δe) − Δc, where λ represents the willingness-to-pay threshold.

### Site-wise and combined analysis plan

While site-wise analyses will provide insights into differences across study sites, results will also be presented for all sites combined and separately for the Asia and Africa regions. Variations in care pathways will be addressed through hospital-level stratification of staff, medicine, and consumable costs, using infant-days as weights to derive site averages. Costs for partial treatment will be estimated at the hospital level by using infant-days as weights for infants receiving partial treatment. Overall costs for enrolled infants will be estimated at the site level using the weighted average of those receiving recommended treatment and those receiving partial treatment. The combined average across all sites will be estimated by using enrolled infants at each site as weights for each treatment arm. The number of infants receiving at least 80% of the recommended treatment in each treatment arm will be used as weights to estimate the combined average hospital costs for the recommended treatment across all sites.

Average weighted inpatient bed costs at the site level will be estimated across all hospitals for each treatment arm using the number of SYIs enrolled in paediatric and newborn wards as weights. Combined inpatient bed costs will then be estimated using the total number of SYIs enrolled in these wards as weights at each site. Average operational costs will also be stratified at the hospital level within each site by using the number of SYIs assessed as weights, and site-stratified for combined analyses using the number of SYIs enrolled as weights at each site.

Average household costs – including registration, food, transport, and wage loss per SYI – will be calculated at the site level using the household survey sample size as the denominator. For pooled analyses, enrolled SYIs will be used as weights across sites. Both the median and the arithmetic mean will be reported to describe the distribution of costs for each variable. Uncertainty in CE will be presented using site-level, regional-level, and pooled ICERs plotted on CE planes. In addition, the mean INB will be estimated and pooled across sites, and 95% confidence intervals will be derived using the *t*-distribution based on site-level variation. An INB greater than zero will indicate that the intervention is cost-effective at the specified willingness-to-pay threshold.

All costs will be presented in 2024 USD. CE results will be interpreted in relation to country-specific willingness-to-pay thresholds and opportunity cost considerations. These findings will provide context-specific evidence to inform national policy decisions rather than relying on outdated GDP-based benchmarks [31].

## DISCUSSION

We present a detailed methodological framework for estimating the costs of managing PSBI in YIs, integrating both health system and household perspectives across different contexts. To our knowledge, we are the first to comprehensively assess both economic and clinical outcomes using such a dual-perspective approach. By analysing medical treatment costs – including staff time, medicines, consumables, inpatient bed costs, and indirect operational costs – as well as non-medical costs such as transport, food, and wage loss, our framework captures the full economic burden faced by hospitals and households. The methodology enables comparison of costs for treating low-mortality-risk PSBI in both hospital and outpatient settings, as well as moderate-mortality-risk PSBI in YIs under shorter and longer hospital stays.

By establishing a standardised approach to CEA using both ICERs and INB, and by incorporating both societal and payer perspectives, the framework supports robust evaluation of RCT outcomes. This approach strengthens evidence-based decision-making and policy formulation. The findings from this study have the potential to reshape PSBI management guidelines and support a paradigm shift in PSBI care. If outpatient treatment for YIs with low-mortality-risk PSBI signs and shorter hospital stays for moderate-mortality-risk cases are shown to be safe and cost-effective, these strategies could substantially reduce both household and health system costs while improving access to care in resource-constrained settings. Policymakers, providers, and payers would benefit from these insights.

Current WHO PSBI treatment guidelines [24] recommend seven days of hospitalisation with injectable antibiotics for YIs presenting with any sign of PSBI. However, in many low- and middle-income countries (LMICs), access to care remains limited and adherence to referral recommendations is low due to financial, logistical, or cultural barriers. Prolonged hospital stays impose a significant economic burden on families and exacerbate resource constraints for already overburdened health systems in LMICs. By integrating both economic and clinical evidence, this study contributes to the broader goal of improving neonatal survival and equity in healthcare access in resource-constrained settings. The findings will inform discussions of the WHO guideline [24] by translating trial-based costs into routine health system settings, excluding research costs and adjusting for adherence. Budget impact analyses can further estimate the financial implications of scaling up these interventions under different coverage scenarios.

### Strengths and limitations

We integrate economic and clinical evidence to identify safe, cost-efficient treatment strategies that could reduce economic burdens, inform national budgeting decisions, and potentially support revisions to WHO guidelines for improved infant care in low-resource settings worldwide.

A key strength is that the analysis is embedded within a multi-country RCT and incorporates both provider and household perspectives to assess CE from societal and provider/payer perspectives. The use of site-level stratification and INB methods further enhances transparency in quantifying uncertainty and supports decision-making using threshold-based interpretation.

Hospital cost data will be collected from all hospitals that randomise infants into treatment arms and aggregated across sites. Provider and household cost data will be collected over a consistent, recent financial period, ensuring alignment of price inputs and minimising variability due to inflation or exchange rate fluctuations during the multi-year enrolment period. Household expenditures will be collected close to the time of care (day eight, 15, or within two weeks after the 15-day outcome visit), with cross-verification against receipts or facility records where available, thereby reducing recall bias. Provider cost and outcome analyses will include the full randomised cohort, strengthening the robustness of the primary CE estimates.

One limitation is that household costs and wage data will be collected during the final nine months of the study using a purposive sample of approximately 10–15% of enrolled infants in each treatment arm at each site. Although this improves operational feasibility, it may introduce selection bias when estimating non-medical costs if respondents differ systematically from those not interviewed. No imputation or adjustment methods will be applied; although missing data are assumed to be random, non-random patterns cannot be entirely ruled out.

The analysis does not account for broader health system constraints, implementation barriers, capacity limitations, or informal healthcare costs that families may incur. The impact inventory focuses on direct effects within the healthcare sector and does not include impacts on other sectors. Factors such as supply chain inefficiencies and quality-of-care metrics specific to PSBI treatment are also outside the scope of this framework. In addition, research-related costs, including staff time for outcome assessment, monitoring and supervision, and protocol-specific training, will not be included.

The study period varies across hospitals and sites. While outcome data will be collected throughout the study, cost data will be collected during the final nine months. Although this may raise concerns regarding representativeness and recall bias, all cost data will be collected in local currency using a consistent price year, ensuring that salaries, medicine prices, and other treatment cost components remain constant for the defined price year.

Despite these limitations, this framework provides robust and policy-relevant evidence for evaluating cost-effective strategies to improve the management of PSBI in YIs in resource-constrained settings.

## CONCLUSIONS

This study presents a comprehensive methodological framework for evaluating the cost-effectiveness of alternative treatment strategies for PSBI in YI across Asia and Africa. By integrating both health system and household perspectives, the framework captures the broader economic implications of outpatient treatment and shorter hospitalisation strategies in resource-constrained settings. The use of standardised costing methods, ICER and incremental net benefit approaches, and multi-country randomised trial data strengthens the robustness and policy relevance of the analysis. Findings from this work will support evidence-based decisions for optimising PSBI management and may contribute to future revisions of WHO guidelines to improve access, affordability, and neonatal outcomes globally.

## Additional material


Online Supplementary Document
