# Supplementary material for: Evaluating the cost-effectiveness of new treatment strategies for the management of young infants with low- or moderate-mortality risk signs of possible serious bacterial infection: framework and study methodology for randomised controlled trials in six countries across Africa and Asia
Source: J Glob Health. 2026 Jun 26;16:05003. doi: 10.7189/jogh.16.05003 (PMC13307443; doi:10.7189/jogh.16.05003)
Supplement: Online Supplementary Document [file jogh-16-05003-s001.pdf]

Supplement to: Garg CC, Nisar YB; PSBI Cost-Effectiveness Study Group. Evaluating the cost-effectiveness of new treatment strategies for the management of young infants with low- or moderate-mortality risk signs of possible serious bacterial infection: framework and study methodology for randomised controlled trials in six countries across Africa and Asia. J Glob Health. 2026;16:05003.

Table S1: CHEERS 2022 Checklist

	Item	Guidance for Reporting	Reported in section
**TITLE**			
Title	1	Identify the study as an economic evaluation and specify the interventions being compared.	Yes. Title identifies study as economic evaluation Specific Interventions reported in introduction and methods section. On page 6.
**ABSTRACT**			
Abstract	2	Provide a structured summary that highlights context, key methods, results and alternative analyses.	Highlights context, key methods, analysis and conclusions (Page 4). It's a methods paper, so no results are presented.
**INTRODUCTION**			
Background and objectives	3	Give the context for the study, the study question and its practical relevance for decision making in policy or practice.	Yes. In introduction. Pages 6-7. Objectives clearly describe comparison of inpatient vs outpatient strategies and shorter vs longer hospitalization.
**METHODS**			
Health economic analysis plan	4	Indicate whether a health economic analysis plan was developed and where available.	This paper is for the analysis plan. Trial-based cost-effectiveness framework following CHEERS 2022 guidance.
Study population	5	Describe characteristics of the study population (such as age range, demographics, socioeconomic, or clinical characteristics).	Young infants (0–59 days) with low- or moderate-mortality-risk PSBI signs More details available in published papers- at https://www.sciencedirect.com/science/article/pii/S2214109X25002438 Optimal place of treatment for young infants aged less than two months with any low-mortality-risk sign of possible serious bacterial infection: Study Protocol for a randomised controlled trial from low- and middle-income countries — JOGH Inpatient versus outpatient management of young infants with a single low-mortality-risk sign of possible serious bacterial infection in sub-Saharan Africa and south Asia: an open-label, multicentre, two-arm, randomised controlled trial - The Lancet Global Health
Setting and location	6	Provide relevant contextual information that may influence findings.	Yes, In methods- study design and participants. - Multi-country RCTs in Bangladesh, Ethiopia, India (two sites), Nigeria, Pakistan, Tanzania. On Page 8
Comparators	7	Describe the interventions or strategies being compared and why chosen.	Yes. Methods- interventions and control. On Page# 8 and 10-11
Perspective	8	State the perspective(s) adopted by the study and why chosen.	Yes. Both Societal and payer/ provider perspective will be considered with intention to treat approach. Payer perspective will also be considered for per protocol analysis for sensitivity. This

			follows the guidance in Recommendations for Conduct, Methodological Practices, and Reporting of Cost-effectiveness Analyses: Second Panel on Cost-Effectiveness in Health and Medicine. JAMA. 2016
Time horizon	9	State the time horizon for the study and why appropriate.	Costs evaluated during the treatment period upto 7 days; outcomes up to day 15 post-treatment for SYI. Overall study period 24 June 2021-19 August 2024.reported on Page#10
Discount rate	10	Report the discount rate(s) and reason chosen.	Not chosen. Costs will be derived for 2024 US dollars (page #14-15)
Selection of outcomes	11	Describe what outcomes were used as the measure(s) of benefit(s) and harm(s).	Poor clinical outcomes outlined on page# 9
Measurement of outcomes	12	Describe how outcomes used to capture benefit(s) and harm(s) were measured.	Used mean effectiveness rates – derived based on non-poor outcomes in total SYI in the group treated. On page 9-10
Valuation of outcomes	13	Describe the population and methods used to measure and value outcomes.	On page 9-10
Measurement and valuation of resources and costs	14	Describe how costs were valued.	Under cost framework, data collection and statistical analysis. Pages 11-25
Currency, price date, and conversion	15	Report the dates of the estimated resource quantities and unit costs, plus the currency and year of conversion.	Overall study period 24 June 2021-19 August 2024.reported on Page#10. Currency mentioned on Page#15
Rationale and description of model	16	If modelling is used, describe in detail and why used. Report if the model is publicly available and where it can be accessed.	Cost- effectiveness planes on pages 26-28
Analytics and assumptions	17	Describe any methods for analysing or statistically transforming data, any extrapolation methods, and approaches for validating any model used.	Weighted averages will be used . No extrapolation methods or models will be used.
Characterizing heterogeneity	18	Describe any methods used for estimating how the results of the study vary for sub-groups.	Yes, CEA planes - sites and region specific and combined analysis. Analysis will be at site, regional and combined level . Page # 28
Characterizing distributional effects	19	Describe how impacts are distributed across different individuals or adjustments made to reflect priority populations.	Costs and Outcomes considered for all enrolled individuals and those receiving 80% treatment. Also both median and mean will be estimated with 95% confidence interval for characterising distribution.
Characterizing uncertainty	20	Describe methods to characterize any sources of uncertainty in the analysis.	95% confidence intervals for presentation of results. Explained on page 18.
Approach to engagement with patients and others affected by the study	21	Describe any approaches to engage patients or service recipients, the general public, communities, or stakeholders (e.g., clinicians or payers) in the design of the study.	Not involved in design per se, but informed consent sought from providers and households.
**RESULTS**			
Study parameters	22	Report all analytic inputs (e.g., values, ranges, references) including uncertainty or distributional assumptions.	Actual results not presented. Only methods
Summary of main results	23	Report the mean values for the main categories of costs and outcomes of interest and summarise them in the most appropriate overall measure.	Actual results not presented. Only methods
Effect of	24	Describe how uncertainty about analytic judgments, inputs, or projections	Uncertainty will be captured through 95% confidence intervals using t values, which

uncertainty		affect findings. Report the effect of choice of discount rate and time horizon, if applicable.	account for skewness and heterogeneous variance for small samples. No discount rate used. Explained on page 18.
Effect of engagement with patients and others affected by the study	25	Report on any difference patient/service recipient, general public, community, or stakeholder involvement made to the approach or findings of the study	Findings will not be discussed with the patients. Only with the site-specific research staff.
**DISCUSSION**			
Study findings, limitations, generalizability, and current knowledge	26	Report key findings, limitations, ethical or equity considerations not captured, and how these could impact patients, policy, or practice.	Yes. Reported in the conclusion section on pages 29-31
**OTHER RELEVANT INFORMATION**			
Source of funding	27	Describe how the study was funded and any role of the funder in the identification, design, conduct, and reporting of the analysis	Funding agency and Statement on Page 33.
Conflicts of interest	28	Report authors conflicts of interest according to journal or International Committee of Medical Journal Editors requirements.	Yes. On page 34.

Husereau D, Drummond M, Augustovski F, de Bekker-Grob E, Briggs AH, Carswell C, Caulley L, Chaiyakunapruk N, Greenberg D, Loder E, Mauskopf J, Mullins CD, Petrou S, Pwu RF, Staniszewska S; CHEERS 2022 ISPOR Good Research Practices Task Force. Consolidated Health Economic Evaluation Reporting Standards 2022 (CHEERS 2022) Statement: Updated Reporting Guidance for Health Economic Evaluations. BMJ. 2022;376:e067975.
The checklist is Open Access distributed in accordance with the terms of the Creative Commons Attribution (CC BY 4.0) license, which permits others to distribute, remix, adapt and build upon this work, for commercial use, provided the original work is properly cited. See: http://creativecommons.org/licenses/by/4.0/.

PSBI Cost-Effectiveness Study Group members:

Members from Bangladesh are: Abdullah H Baqui, Johns Hopkins Bloomberg School of Public Health, Baltimore, USA; Mohammod Shahidullah, Bangabandhu Sheikh Mujib Medical University, Dhaka, Bangladesh; Salahuddin Ahmed, Projahnmo Research Foundation, Dhaka, Bangladesh; Arunangshu Dutta Roy, Projahnmo Research Foundation, Dhaka, Bangladesh; Rasheda Khanam, Johns Hopkins Bloomberg School of Public Health, Baltimore, USA; Iffat Ara Jaben, Projahnmo Research Foundation, Dhaka, Bangladesh; Nabidul Haque Chowdhury, Projahnmo Research Foundation, Dhaka, Bangladesh; Kisholoy Choudhury, Projahnmo Research Foundation, Dhaka, Bangladesh; Sabina Ashrafee Lipi, National Newborn Health Program, Directorate General of Health Services, Dhaka, Bangladesh; Md Jahurul Islam, National Newborn Health Program, Directorate General of Health Services, Dhaka, Bangladesh; Manajjir Ali, Projahnmo Research Foundation, Dhaka, Bangladesh.

Members from Ethiopia are: Amha Mekasha, Addis Ababa University, Addis Ababa, Ethiopia; Lulu Muhe, Addis Ababa University, Addis Ababa, Ethiopia; Damen Hailemariam, Addis Ababa University, Addis Ababa, Ethiopia; Dorka Woldesenbet Keraga, Addis Ababa University, Addis Ababa, Ethiopia; Tabot Keskis Azeze, Addis Ababa University, Addis Ababa, Ethiopia; Abiy Seifu Estifanos, Center for Implementation Sciences in Health, Aklilu Lemma Institute of Health Research, Addis Ababa University, Ethiopia; Bogale Worku, Ethiopian Pediatric Society, Addis Ababa, Ethiopia; Solome Jebessa, St. Paul's Hospital Millennium Medical College, Addis Ababa, Ethiopia.

Members from India are: Archana Thakur, Society for Applied Studies, New Delhi, India; Temsunaro Rongsen-Chandola, Society for Applied Studies, New Delhi, India; Nidhi Goyal, Society for Applied Studies, New Delhi, India; Amit Kumar, Society for Applied Studies, New Delhi, India; Nita Bhandari, Society for Applied Studies, New Delhi, India; Uma Chandra Mouli Natchu, Society for Applied Studies, New Delhi, India; Manisha Gupta, Society for Applied Studies, New Delhi, India; Aritra Guha, Society for Applied Studies, New Delhi, India; Shayam Kaushik, YS Parmar Government Medical College, Nahan, Himachal Pradesh, India; Surjeet Kumar, YS Parmar Government Medical College, Nahan, Himachal Pradesh, India; Amitabh Jain, Civil Hospital Paonta Sahib, Sirmaur, Himachal Pradesh, India; Jagjit Singh Dalal, Pt. BD Sharma Post Graduate Institute of Medical Sciences, Rohtak, Haryana, India; Kundan Mittal, Pt. BD Sharma Post Graduate Institute of Medical Sciences, Rohtak, Haryana, India; GP Kaushal, B.S. Ambedkar Hospital, Rohini, New Delhi, India; Vineeta Wadhwa, B.S. Ambedkar Hospital, Rohini, New Delhi, India; Anju Seth, Lady Hardinge Medical College and Associated Hospitals, New Delhi, India; Varinder Singh, Lady Hardinge Medical College and Associated Hospitals, New Delhi, India; Harish Pemde, Lady Hardinge Medical College and Associated Hospitals, New Delhi, India; Praveen Kumar, Lady Hardinge Medical College and Associated Hospitals, New Delhi, India; Viswas Chhapola, Lady Hardinge Medical College and Associated Hospitals, New Delhi, India; Yashwant Kumar Rao, GSVM Medical College, Kanpur, India; Arun Kumar Arya, GSVM Medical College, Kanpur, India; Krishna Kumar Dokania, Shyam Children & Maternity Centre, Kanpur, India; Pankaj Kumar, Government of Uttar Pradesh, India; Ved Prakash, Directorate of

Medical & Health Services, Government of Uttar Pradesh, India; Amit Singh, Directorate of Medical & Health Services, Government of Uttar Pradesh, India; Suryanshu Ojha, National Health Mission, Government of Uttar Pradesh, India; Shakal Narayan Singh, King George's Medical University, Lucknow, India; Neeraj Kumar, SN Medical College, Agra, India; Shiv Kumar, AHM & Dufferin District Women's Hospital, Kanpur, India; Vinay Pratap Singh, Community Empowerment Laboratory, Lucknow, India; Malvika Mishra, Community Empowerment Laboratory, Lucknow, India; Pramod Kumar Singh, Community Empowerment Laboratory, Lucknow, India; Vivek Kumar Singh, Community Empowerment Laboratory, Lucknow, India; Amit Tandon, Community Empowerment Laboratory, Lucknow, India; Saumya Dwivedi, Community Empowerment Laboratory, Lucknow, India; Priya Chaturvedi, Community Empowerment Laboratory, Lucknow, India; Madhuri Tiwari, Community Empowerment Laboratory, Lucknow, India; Rashmi Kumar, Community Empowerment Laboratory, Lucknow, India; Aarti Kumar, Community Empowerment Laboratory, Lucknow, India; Vishwajeet Kumar, Community Empowerment Laboratory, Lucknow, India.

Members from Nigeria are: Robinson Daniel Wammanda, Department of Paediatrics, Ahmadu Bello University Teaching Hospital, Zaria, Nigeria; Laila Hassan, Department of Paediatrics, Ahmadu Bello University Teaching Hospital, Zaria, Nigeria; Ishaku Hassan, Department of Paediatrics, Ahmadu Bello University Teaching Hospital, Zaria, Nigeria; Saraja Ahmodu Opaluwa, Department of Medical Microbiology, Ahmadu Bello University Teaching Hospital, Zaria, Nigeria; Bawa Ega, Department of Medical Microbiology, Ahmadu Bello University Teaching Hospital, Zaria, Nigeria; Aminu Shadrach Adamu, Department of Community Medicine, Ahmadu Bello University Teaching Hospital, Zaria, Nigeria; Daniel Efemena Atinaya, African Neonatal Sepsis Trial Research Unit, Ahmadu Bello University Teaching Hospital, Zaria, Nigeria.

Members from Pakistan are: Fyezah Jehan, Aga Khan University, Karachi, Pakistan; Imran Nisar, Aga Khan University, Karachi, Pakistan; Benazir Baloch, Aga Khan University, Karachi, Pakistan; Dania Omer Ansari, Aga Khan University, Karachi, Pakistan; Kiran Lalani, Aga Khan University, Karachi, Pakistan; Najeeb Rehman, Aga Khan University, Karachi, Pakistan; Azhar Raza, Aga Khan University, Karachi, Pakistan; Tooba Ahmed Alvi, Aga Khan University, Karachi, Pakistan; Salman Osmani, Aga Khan University, Karachi, Pakistan; Aneeta Hotwani, Aga Khan University, Karachi, Pakistan; Fatimah Azhar, Aga Khan University, Karachi, Pakistan.

Members from Tanzania are: Karim Manji, Department of Pediatrics, Muhimbili University of Health and Allied Sciences, Dar-es-Salaam, Tanzania; Rodrick Kisenge, Department of Pediatrics, Muhimbili University of Health and Allied Sciences, Dar-es-Salaam, Tanzania; Raban Rameck, Department of Pediatrics, Muhimbili University of Health and Allied Sciences, Dar-es-Salaam, Tanzania; Nahya Salim, Department of Pediatrics, Muhimbili University of Health and Allied Sciences, Dar-es-Salaam, Tanzania; Sarah Somji, Department of Pediatrics, Muhimbili University of Health and Allied Sciences, Dar-es-Salaam, Tanzania; Mohamed Kheri Bakari, Department of Pediatrics, Muhimbili University of Health and Allied Sciences, Dar-es-Salaam, Tanzania; Fatimah Dhallah, Department of Pediatrics, Muhimbili University of Health

and Allied Sciences, Dar-es-Salaam, Tanzania; Fred Maleko, Department of Pediatrics, Muhimbili University of Health and Allied Sciences, Dar-es-Salaam, Tanzania; Kristina Lugangira, Department of Pediatrics, Muhimbili University of Health and Allied Sciences, Dar-es-Salaam, Tanzania; Veneranda M Ndensangia, Department of Pediatrics, Muhimbili University of Health and Allied Sciences, Dar-es-Salaam, Tanzania; Christopher R. Sudfeld, Harvard T.H. Chan School of Public Health, Boston, USA; Christopher P Duggan, Boston Children's Hospital, Boston, USA.

Members from the USA are: Divya D Bhasin, Syzygy Consulting LLC, USA; Nikita Bindra, Syzygy Consulting LLC, USA; Karina Gupta, Syzygy Consulting LLC, USA; Chandrika Garg, Syzygy Consulting LLC, California, USA; Yasir Bin Nasir, Department of Sexual, Reproductive, Maternal, Child and Adolescent and Ageing Health, World Health Organization, Geneva, Switzerland; Shamim A Qazi, Independent Newborn and Child Consultant, Switzerland
